# Crystal structure of bis­[1-(naphthalen-1-ylmeth­yl)pyridinium] bis­(2,2-di­cyano­ethene-1,1-di­thiol­ato-κ^2^ *S*,*S*′)nickelate(II)

**DOI:** 10.1107/S1600536814017012

**Published:** 2014-07-31

**Authors:** Miao Zhang, Xu-Jie Xiong

**Affiliations:** a College of Chemical Engineering, Huanggang Normal University, 438000 Huangzhou, People’s Republic of China; b Hubei Key Laboratory for Processing and Application of Catalytic Materials, Huanggang Normal University, 438000 Huangzhou, People’s Republic of China

**Keywords:** bis­(2,2-bi­cyano­ethene-1,1-di­thiol­ato)nickel(II), pyridinium, hydrogen bonding, π–π inter­action, crystal structure.

## Abstract

A new ion-pair complex, (C_16_H_14_N)_2_[Ni(C_4_N_2_S_2_)_2_] or (1-NaMePy)_2_[Ni(imnt)_2_], where 1-NaMePy is 1-(4-naphthyl­methyl­ene)pyridinium and imnt is 2,2-di­cyano­ethene-1,1-di­thiol­ate, was obtained by the direct reaction of NiCl_2_, K_2_imnt and (1-NaMePy)^+^Br^−^ in H_2_O. The asymmetric unit contains a [1-NaMePy]^+^ cation and one half of an Ni(imnt)_2_^2−^ anion. The Ni^II^ ion lies on an inversion centre and adopts a square-planar configuration with Ni—S bond lengths of 2.200 (1) and 2.216 (1) Å. In the [1-NaMePy]^+^ cation, the naphthyl ringsystem and the pyridinium ring make a dihedral angle of 90.0 (2)°. In the crystal, C—H⋯N and C—H⋯Ni hydrogen bonds, as well as π–π inter­actions between the chelate ring and the pyridinium ring [centroid–centroid distance = 3.675 (2) Å] link the ions into a three-dimensional network.

## Abstract 2

In the ion-pair complex, bis­[1-(naphthalen-1-ylmeth­yl)pyridinium] bis­(2,2-di­cyano­ethene-1,1-di­thiol­ate-κ^2^ *S*,*S*′)nickelate(II), C—H⋯N and C—H⋯Ni hydrogen bonds as well as π–π inter­actions between the ions result in the formation of a three-dimensional network.

## Chemical context   

Transition metal complexes with di­thiol­ate ligands such as 2,2-di­cyano­ethene-1,1-di­thiol­ate (imnt) or 1,2-di­cyano­ethene-1,2-di­thiol­ate (mnt) are important mol­ecular materials with inter­esting electrical conductivity, superconductivity, optical and magnetic properties (Liu *et al.*, 1996 ▶; Robertson & Cronin, 2002 ▶; Ni *et al.*, 2005 ▶; Ren *et al.*, 2002 ▶; Xie *et al.*, 2002 ▶; Duan *et al.* 2010 ▶). Recently, attempts have been made to extend the range of metal complexes containing the Ni(imnt)_2_^2−^ anion, and the topology and the size of some organic cations, such as substituted benzyl pyridinium derivatives, play an important role in tuning the stacks of anions and cations of mol­ecular materials containing the Ni(imnt)_2_^2−^ anion (Liu *et al.*, 2006 ▶; Feng *et al.*, 2007 ▶). The title ion-pair complex, (1-NaMePy)_2_[Ni(imnt)_2_] has therefore been prepared and investigated. 
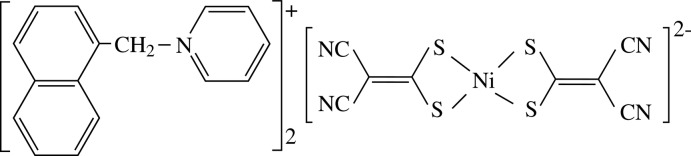


## Structural commentary   

The asymmetric unit of the title compound consists of one [1-NaMePy]^+^ cation and one-half of an Ni(imnt)_2_^2−^ anion located about an inversion center. The NiS_4_ core exhibits a square-planar configuration, with Ni—S bond lengths of 2.200 (1) and 2.216 (1) Å. The S1—Ni1—S2 bond angle within the four-membered ring (Ni1/S1/C1/S2) is 78.91 (3)°. The N1 and N2 atoms of the C N groups deviate from the Ni1/S1/C1/S2 plane by 0.078 (3) and 0.169 (3) Å, respectively. The [1-NaMePy]^+^ cation adopts a conformation in which both the naphthyl ring system and the pyridinium ring are twisted with respect to the N3/C11/C10 reference plane, making dihedral angles of 10.5 (2)° and 87.3 (3)°, respectively. The naphthyl ring system and the pyridinium ring make a dihedral angle of 90.0 (2)°.

## Supra­molecular features   

**Figure 2:**
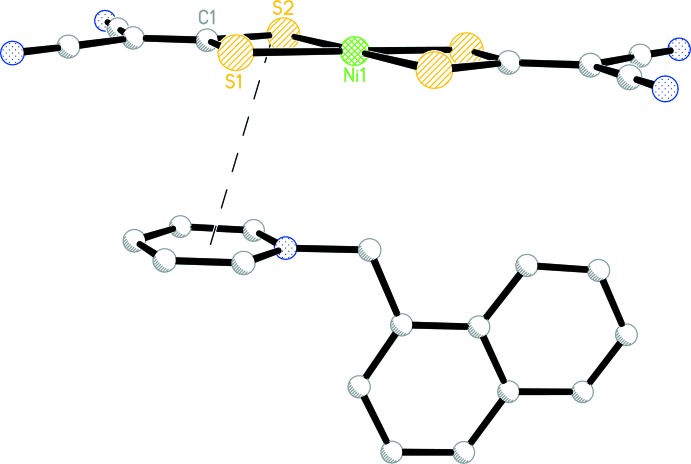
The π–π contact between the chelate ring of the anion and the pyridinium ring of the cation (shown as a dashed line).

**Figure 3:**
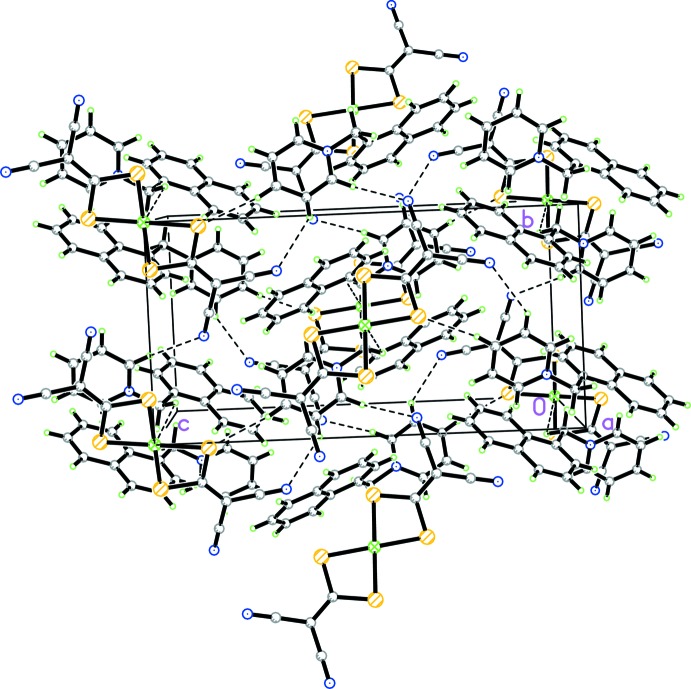
The packing of the title compound, viewed down the *a* axis, showing the network of mol­ecules connected by C—H⋯N hydrogen bonds (dashed lines).

There are three weak inter­actions between the Ni(imnt)_2_^2−^ anion and [1-NaMePy]^+^ cation. The first is a π–π contact between the chelate ring (which is defined by atoms Ni1, S1, S2, and C1) of the anion and the pyridinium ring of the cation (Fig. 2 ▶) with a distance of 3.675 (2) Å between the centroids. The second is a C—H⋯Ni hydrogen bond and the third is a C—H⋯N hydrogen bond (Table 1 ▶, Fig. 3 ▶). The combination of these weak inter­actions consolidates the title complex into a three-dimensional network structure (Fig. 3 ▶).

## Database survey   

Many ion-pair complexes containing Ni(imnt)_2_^2−^ anion have been reported, typical examples being [TBA]_2_[Ni(imnt)_2_] and [4NO_2_BzPy]_2_[Ni(imnt)_2_] [TBA is tetra­butyl­ammonium; 4NO_2_BzPy is 1-(4-nitro­benz­yl)pyridinium] (Liu *et al.*, 2006 ▶), [4FBzPy]_2_[Ni(imnt)_2_] [4FBzPy is 1-(4-fluoro­benz­yl)pyrid­in­ium] (Zhou & Ni, 2007 ▶), [Bz2NH_2_Py]_2_[Ni(imnt)_2_] (Bz2NH_2_Py is 1-benzyl-2-amino­pyridinium) (Hou *et al.*, 2007 ▶), [BzDMAP]_2_[Ni(imnt)_2_] [BzDMAP is 1-benzyl-4-(di­methyl­amino)­pyridinium] (Feng *et al.*, 2007 ▶), [2-NaMePy]_2_[Ni(imnt)_2_] and [2-NaMe-4-MePy]_2_[Ni(imnt)_2_] [2-NaMePy is 1-(2-naphthyl­meth­yl)pyridinium; 2-NaMe-4-MePy is 1-(2-naphthyl­meth­yl)-4-methyl­pyridinium] (Huang *et al.*, 2009 ▶), [Bz-4-MePy]_2_[Ni(imnt)_2_] and [Bz-4-MeQl]_2_[Ni(imnt)_2_] (Bz-4-MePy is 1-benzyl-4-methyl­pyridinium; Bz-4-MeQl is 1-benzyl-4-methyl­quinolinium) (Liu *et al.*, 2013 ▶). For a description of C—H⋯N and C—H⋯Ni hydrogen bonds, see: Huang *et al.*, (2009 ▶). For a description of π–π contacts between chelate and phenyl rings, see: Molčanov *et al.* (2013 ▶).

## Synthesis and crystallization   

The title ion-pair complex was prepared by the direct reaction of 1:2:2 mol equiv. of NiCl_2_·6H_2_O, K_2_imnt and 1-(4-naphthyl­methyl­ene)pyridinium bromide in water (Huang *et al.*, 2009 ▶). The brown product obtained was purified through recrystallization from a mixed solvent of methanol and water (yield: 78%). Brown block-shaped single crystals suitable for X-ray analysis were obtained by slow evaporation of a methanol solution at room temperature after about 4 weeks.

## Refinement   

All H-atoms were positioned geometrically and refined using a riding model with *d*(C—H) = 0.93 Å, *U*_iso_(H) = 1.2*U*_eq_(C) for aromatic and *d*(C—H) = 0.97 Å, *U*_iso_(H) = 1.2*U*_eq_(C) for CH_2_ atoms. Crystal data, data collection and structure refinement details are summarized in Table 2 ▶.

## Supplementary Material

Crystal structure: contains datablock(s) I, global. DOI: 10.1107/S1600536814017012/kp2472sup1.cif

Structure factors: contains datablock(s) I. DOI: 10.1107/S1600536814017012/kp2472Isup2.hkl

Supporting information file. DOI: 10.1107/S1600536814017012/kp2472Isup3.cml

CCDC reference: 1015644

Additional supporting information:  crystallographic information; 3D view; checkCIF report

## Figures and Tables

**Figure 1:**
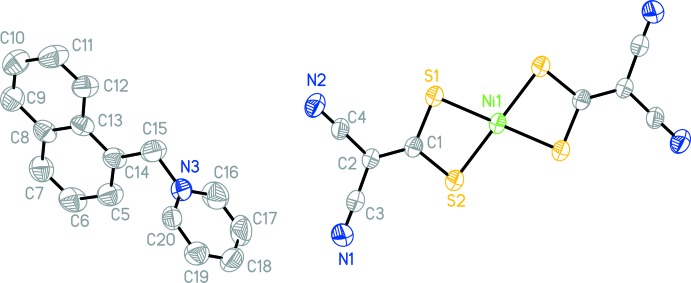
The mol­ecular structure of (I), with the atom labelling and 30% probability displacement ellipsoids for non-H atoms The other half of the anion is generated by the inversion-symmetry operation −*x*, *y* + 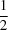, −*z* + 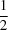.

**Table 1:** Hydrogen-bond geometry (Å, °)

*D*—H⋯*A*	*D*—H	H⋯*A*	*D*⋯*A*	*D*—H⋯*A*
C19—H19⋯N2^i^	0.93	2.60	3.265 (5)	129
C20—H20⋯N1^ii^	0.93	2.42	3.304 (5)	160
C15—H15*B*⋯Ni1^iii^	0.97	3.07	3.508 (4)	109

Symmetry codes: (i) 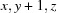; (ii) 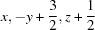; (iii) 
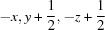
.

**Table 2:** Experimental details

Crystal data	
Chemical formula	(C_16_H_14_N)_2_[Ni(C_4_N_2_S_2_)_2_]
*M*_r_	779.63
Crystal system, space group	Monoclinic, *P*2_1_/*c*
Temperature (K)	291
*a*, *b*, *c* (Å)	11.876 (3), 9.025 (3), 17.465 (5)
β (°)	91.808 (4)
*V* (Å^3^)	1871.0 (9)
*Z*	2
Radiation type	Mo *K*α
μ (mm^−1^)	0.78
Crystal size (mm)	0.36 × 0.30 × 0.21

Data collection	
Diffractometer	Bruker *SMART* CCD area detector
Absorption correction	Multi-scan (*SADABS*; Bruker, 2000 ▶)
*T*_min_, *T*_max_	0.762, 0.843
No. of measured, independent and observed [*I* > 2σ(*I*)] reflections	9345, 3283, 2228
*R*_int_	0.031
(sin θ/λ)_max_ (Å^−1^)	0.595

Refinement	
*R*[*F* ^2^ > 2σ(*F* ^2^)], *wR*(*F* ^2^), *S*	0.040, 0.102, 1.04
No. of reflections	3283
No. of parameters	232
H-atom treatment	H-atom parameters constrained
Δρ_max_, Δρ_min_ (e Å^−3^)	0.21, −0.15

Computer programs: *SMART* and *SAINT* (Bruker, 2000 ▶) and *SHELXTL* (Sheldrick, 2008 ▶).

## supplementary crystallographic information

### Crystal data

**Table:**

(C_16_H_14_N)_2_[Ni(C_4_N_2_S_2_)_2_]	*F*(000) = 804
*M**_r_* = 779.63	*D*_x_ = 1.384 Mg m^−^^3^
Monoclinic, *P*2_1_/*c*	Mo *K*α radiation, λ = 0.71073 Å
Hall symbol: -P 2ybc	Cell parameters from 1994 reflections
*a* = 11.876 (3) Å	θ = 2.5–22.7°
*b* = 9.025 (3) Å	µ = 0.78 mm^−^^1^
*c* = 17.465 (5) Å	*T* = 291 K
β = 91.808 (4)°	Block, brown
*V* = 1871.0 (9) Å^3^	0.36 × 0.30 × 0.21 mm
*Z* = 2	

### Data collection

**Table:**

Bruker SMART CCD area detector diffractometer	3283 independent reflections
Radiation source: fine-focus sealed tube	2228 reflections with *I* > 2σ(*I*)
Graphite monochromator	*R*_int_ = 0.031
φ and ω scans	θ_max_ = 25.0°, θ_min_ = 2.3°
Absorption correction: multi-scan (*SADABS*; Bruker, 2000)	*h* = −13→14
*T*_min_ = 0.762, *T*_max_ = 0.843	*k* = −9→10
9345 measured reflections	*l* = −20→20

### Refinement

**Table:**

Refinement on *F*^2^	Primary atom site location: structure-invariant direct methods
Least-squares matrix: full	Secondary atom site location: difference Fourier map
*R*[*F*^2^ > 2σ(*F*^2^)] = 0.040	Hydrogen site location: inferred from neighbouring sites
*wR*(*F*^2^) = 0.102	H-atom parameters constrained
*S* = 1.04	*w* = 1/[σ^2^(*F*_o_^2^) + (0.046*P*)^2^ + 0.1262*P*] where *P* = (*F*_o_^2^ + 2*F*_c_^2^)/3
3283 reflections	(Δ/σ)_max_ < 0.001
232 parameters	Δρ_max_ = 0.21 e Å^−^^3^
0 restraints	Δρ_min_ = −0.15 e Å^−^^3^

### Special details

**Table:**

Geometry. All e.s.d.'s (except the e.s.d. in the dihedral angle between two l.s. planes) are estimated using the full covariance matrix. The cell e.s.d.'s are taken into account individually in the estimation of e.s.d.'s in distances, angles and torsion angles; correlations between e.s.d.'s in cell parameters are only used when they are defined by crystal symmetry. An approximate (isotropic) treatment of cell e.s.d.'s is used for estimating e.s.d.'s involving l.s. planes.
Refinement. Refinement of *F*^2^ against ALL reflections. The weighted *R*-factor *wR* and goodness of fit *S* are based on *F*^2^, conventional *R*-factors *R* are based on *F*, with *F* set to zero for negative *F*^2^. The threshold expression of *F*^2^ > σ(*F*^2^) is used only for calculating *R*-factors(gt) *etc*. and is not relevant to the choice of reflections for refinement. *R*-factors based on *F*^2^ are statistically about twice as large as those based on *F*, and *R*- factors based on ALL data will be even larger.

### Fractional atomic coordinates and isotropic or equivalent isotropic displacement parameters (Å^2^)

**Table:**

	*x*	*y*	*z*	*U*_iso_*/*U*_eq_	
Ni1	0.0000	0.0000	0.0000	0.06043 (19)	
S1	0.03025 (7)	0.02841 (8)	0.12405 (4)	0.0697 (2)	
S2	0.08762 (7)	0.21679 (9)	0.00355 (4)	0.0709 (2)	
N1	0.2232 (3)	0.5408 (4)	0.11604 (17)	0.1156 (12)	
N2	0.1247 (3)	0.2483 (3)	0.29836 (16)	0.0963 (9)	
N3	0.2381 (2)	0.7148 (3)	0.42980 (16)	0.0805 (7)	
C1	0.0909 (2)	0.1950 (3)	0.10140 (14)	0.0616 (7)	
C2	0.1329 (2)	0.2957 (3)	0.15460 (14)	0.0628 (7)	
C3	0.1819 (3)	0.4313 (4)	0.13254 (16)	0.0792 (9)	
C4	0.1274 (3)	0.2685 (3)	0.23417 (17)	0.0704 (8)	
C5	0.4644 (3)	0.7646 (4)	0.4864 (2)	0.1009 (11)	
H5	0.4408	0.7981	0.4382	0.121*	
C6	0.5731 (3)	0.8033 (5)	0.5164 (3)	0.1080 (13)	
H6	0.6212	0.8604	0.4875	0.130*	
C7	0.6062 (3)	0.7565 (4)	0.5872 (2)	0.1045 (12)	
H7	0.6768	0.7843	0.6069	0.125*	
C8	0.5373 (3)	0.6678 (4)	0.6314 (2)	0.0817 (9)	
C9	0.5710 (3)	0.6177 (4)	0.7054 (2)	0.0983 (11)	
H9	0.6410	0.6467	0.7255	0.118*	
C10	0.5068 (4)	0.5308 (5)	0.7473 (3)	0.1114 (13)	
H10	0.5314	0.4992	0.7956	0.134*	
C11	0.4017 (4)	0.4881 (5)	0.7169 (3)	0.1169 (14)	
H11	0.3563	0.4266	0.7455	0.140*	
C12	0.3642 (3)	0.5342 (4)	0.6470 (2)	0.0957 (11)	
H12	0.2933	0.5047	0.6286	0.115*	
C13	0.4307 (3)	0.6261 (3)	0.6015 (2)	0.0769 (9)	
C14	0.3947 (3)	0.6786 (4)	0.5280 (2)	0.0812 (9)	
C15	0.2770 (3)	0.6348 (4)	0.4999 (2)	0.1096 (13)	
H15A	0.2759	0.5292	0.4895	0.131*	
H15B	0.2246	0.6539	0.5402	0.131*	
C16	0.2514 (4)	0.6590 (5)	0.3620 (3)	0.1198 (14)	
H16	0.2866	0.5676	0.3573	0.144*	
C17	0.2150 (5)	0.7315 (7)	0.2989 (3)	0.1362 (19)	
H17	0.2264	0.6917	0.2506	0.163*	
C18	0.1615 (4)	0.8632 (6)	0.3060 (3)	0.1177 (15)	
H18	0.1346	0.9136	0.2627	0.141*	
C19	0.1477 (3)	0.9203 (4)	0.3761 (3)	0.1004 (11)	
H19	0.1118	1.0109	0.3821	0.120*	
C20	0.1868 (3)	0.8436 (4)	0.43750 (19)	0.0822 (9)	
H20	0.1775	0.8821	0.4863	0.099*	

### Atomic displacement parameters (Å^2^)

**Table:**

	*U*^11^	*U*^22^	*U*^33^	*U*^12^	*U*^13^	*U*^23^
Ni1	0.0607 (3)	0.0696 (4)	0.0503 (3)	0.0003 (3)	−0.0087 (2)	−0.0033 (2)
S1	0.0793 (5)	0.0764 (5)	0.0527 (4)	−0.0091 (4)	−0.0100 (4)	0.0017 (4)
S2	0.0824 (5)	0.0771 (5)	0.0528 (4)	−0.0074 (4)	−0.0057 (4)	−0.0001 (4)
N1	0.173 (3)	0.094 (2)	0.080 (2)	−0.040 (2)	−0.010 (2)	0.0011 (17)
N2	0.130 (3)	0.097 (2)	0.0603 (16)	0.0021 (18)	−0.0146 (16)	−0.0046 (15)
N3	0.0765 (18)	0.083 (2)	0.0818 (19)	−0.0104 (15)	−0.0064 (15)	0.0020 (16)
C1	0.0571 (16)	0.0696 (18)	0.0577 (15)	0.0041 (14)	−0.0062 (13)	−0.0005 (14)
C2	0.0666 (18)	0.0695 (19)	0.0517 (16)	−0.0009 (15)	−0.0079 (13)	−0.0009 (14)
C3	0.100 (3)	0.081 (2)	0.0552 (18)	−0.008 (2)	−0.0128 (17)	−0.0031 (17)
C4	0.076 (2)	0.072 (2)	0.0625 (19)	0.0008 (16)	−0.0157 (15)	−0.0052 (16)
C5	0.079 (2)	0.106 (3)	0.118 (3)	−0.009 (2)	−0.004 (2)	0.026 (2)
C6	0.068 (2)	0.118 (3)	0.139 (4)	−0.026 (2)	0.012 (2)	0.022 (3)
C7	0.079 (3)	0.111 (3)	0.122 (3)	−0.014 (2)	−0.011 (2)	0.005 (3)
C8	0.065 (2)	0.071 (2)	0.109 (3)	−0.0020 (17)	0.005 (2)	−0.004 (2)
C9	0.084 (3)	0.102 (3)	0.108 (3)	0.007 (2)	−0.015 (2)	0.006 (2)
C10	0.103 (3)	0.127 (3)	0.104 (3)	0.017 (3)	−0.005 (3)	0.019 (3)
C11	0.097 (3)	0.120 (3)	0.133 (4)	0.010 (3)	0.003 (3)	0.047 (3)
C12	0.070 (2)	0.100 (3)	0.117 (3)	0.000 (2)	−0.002 (2)	0.028 (2)
C13	0.064 (2)	0.0643 (19)	0.103 (2)	0.0054 (16)	0.0043 (19)	0.0066 (18)
C14	0.063 (2)	0.077 (2)	0.103 (2)	−0.0091 (17)	−0.0005 (18)	0.0121 (19)
C15	0.086 (3)	0.119 (3)	0.122 (3)	−0.025 (2)	−0.024 (2)	0.046 (3)
C16	0.143 (4)	0.103 (3)	0.114 (3)	0.002 (3)	0.019 (3)	−0.020 (3)
C17	0.184 (5)	0.153 (5)	0.073 (3)	−0.048 (4)	0.010 (3)	−0.032 (3)
C18	0.117 (4)	0.140 (4)	0.094 (3)	−0.035 (3)	−0.030 (3)	0.034 (3)
C19	0.095 (3)	0.095 (3)	0.111 (3)	−0.003 (2)	−0.007 (2)	0.012 (3)
C20	0.088 (2)	0.086 (2)	0.072 (2)	−0.013 (2)	−0.0014 (18)	−0.0120 (19)

### Geometric parameters (Å, º)

**Table:**

Ni1—S1^i^	2.2000 (9)	C8—C9	1.415 (5)
Ni1—S1	2.2000 (9)	C9—C10	1.329 (5)
Ni1—S2^i^	2.2160 (9)	C9—H9	0.9300
Ni1—S2	2.2160 (9)	C10—C11	1.395 (6)
S1—C1	1.718 (3)	C10—H10	0.9300
S2—C1	1.719 (3)	C11—C12	1.353 (5)
N1—C3	1.144 (4)	C11—H11	0.9300
N2—C4	1.137 (3)	C12—C13	1.409 (4)
N3—C16	1.300 (5)	C12—H12	0.9300
N3—C20	1.321 (4)	C13—C14	1.420 (4)
N3—C15	1.482 (4)	C14—C15	1.519 (4)
C1—C2	1.382 (4)	C15—H15A	0.9700
C2—C3	1.414 (4)	C15—H15B	0.9700
C2—C4	1.415 (4)	C16—C17	1.342 (6)
C5—C14	1.361 (4)	C16—H16	0.9300
C5—C6	1.421 (5)	C17—C18	1.355 (6)
C5—H5	0.9300	C17—H17	0.9300
C6—C7	1.353 (5)	C18—C19	1.344 (5)
C6—H6	0.9300	C18—H18	0.9300
C7—C8	1.395 (5)	C19—C20	1.346 (5)
C7—H7	0.9300	C19—H19	0.9300
C8—C13	1.406 (4)	C20—H20	0.9300
			
S1^i^—Ni1—S1	180.00 (4)	C9—C10—H10	120.8
S1^i^—Ni1—S2^i^	78.91 (3)	C11—C10—H10	120.8
S1—Ni1—S2^i^	101.09 (3)	C12—C11—C10	121.6 (4)
S1^i^—Ni1—S2	101.09 (3)	C12—C11—H11	119.2
S1—Ni1—S2	78.91 (3)	C10—C11—H11	119.2
S2^i^—Ni1—S2	180.00 (4)	C11—C12—C13	121.1 (4)
C1—S1—Ni1	86.09 (9)	C11—C12—H12	119.5
C1—S2—Ni1	85.56 (10)	C13—C12—H12	119.5
C16—N3—C20	120.2 (3)	C8—C13—C12	117.5 (3)
C16—N3—C15	121.3 (4)	C8—C13—C14	119.2 (3)
C20—N3—C15	118.5 (3)	C12—C13—C14	123.2 (3)
C2—C1—S1	124.5 (2)	C5—C14—C13	120.1 (3)
C2—C1—S2	126.1 (2)	C5—C14—C15	122.9 (3)
S1—C1—S2	109.42 (15)	C13—C14—C15	117.0 (3)
C1—C2—C3	121.9 (2)	N3—C15—C14	113.6 (3)
C1—C2—C4	121.3 (3)	N3—C15—H15A	108.8
C3—C2—C4	116.8 (3)	C14—C15—H15A	108.8
N1—C3—C2	178.5 (4)	N3—C15—H15B	108.8
N2—C4—C2	178.7 (4)	C14—C15—H15B	108.8
C14—C5—C6	120.3 (4)	H15A—C15—H15B	107.7
C14—C5—H5	119.9	N3—C16—C17	120.9 (4)
C6—C5—H5	119.9	N3—C16—H16	119.5
C7—C6—C5	119.6 (3)	C17—C16—H16	119.5
C7—C6—H6	120.2	C16—C17—C18	119.4 (4)
C5—C6—H6	120.2	C16—C17—H17	120.3
C6—C7—C8	121.7 (4)	C18—C17—H17	120.3
C6—C7—H7	119.1	C19—C18—C17	119.4 (4)
C8—C7—H7	119.1	C19—C18—H18	120.3
C7—C8—C13	119.0 (3)	C17—C18—H18	120.3
C7—C8—C9	122.3 (3)	C18—C19—C20	118.7 (4)
C13—C8—C9	118.6 (3)	C18—C19—H19	120.7
C10—C9—C8	122.7 (4)	C20—C19—H19	120.7
C10—C9—H9	118.6	N3—C20—C19	121.3 (3)
C8—C9—H9	118.6	N3—C20—H20	119.3
C9—C10—C11	118.4 (4)	C19—C20—H20	119.3

Symmetry code: (i) −*x*, −*y*, −*z*.

### Hydrogen-bond geometry (Å, º)

**Table:**

*D*—H···*A*	*D*—H	H···*A*	*D*···*A*	*D*—H···*A*
C19—H19···N2^ii^	0.93	2.60	3.265 (5)	129
C20—H20···N1^iii^	0.93	2.42	3.304 (5)	160
C15—H15*B*···Ni1^iv^	0.97	3.07	3.508 (4)	109

Symmetry codes: (ii) *x*, *y*+1, *z*; (iii) *x*, −*y*+3/2, *z*+1/2; (iv) −*x*, *y*+1/2, −*z*+1/2.
